# Assessment of geometrical accuracy of magnetic resonance images for radiation therapy of lung cancers

**DOI:** 10.1120/jacmp.v4i4.2510

**Published:** 2003-09-01

**Authors:** N. Koch, H. H. Liu, L. E. Olsson, E. F. Jackson

**Affiliations:** ^1^ Department of Radiation Physics The University of Texas M. D. Anderson Cancer Center 1515 Holcombe Boulevard Houston Texas 77030; ^2^ Department of Imaging Physics The University of Texas M. D. Anderson Cancer Center 1515 Holcombe Boulevard Houston Texas 77030

**Keywords:** magnetic resonance imaging, image distortion, susceptibility effect, cine imaging, lung tumor, tumor motion

## Abstract

The purpose of this research was to investigate the geometrical accuracy of magnetic resonance (MR) images used in the radiation therapy treatment planning for lung cancer. In this study, the capability of MR imaging to acquire dynamic two‐dimensional images was explored to access the motion of lung tumors. Due to a number of factors, including the use of a large field‐of‐view for the thorax, MR images are particularly subject to geometrical distortions caused by the inhomogeneity and gradient nonlinearity of the magnetic field. To quantify such distortions, we constructed a phantom, which approximated the dimensions of the upper thorax and included two air cavities. Evenly spaced vials containing contrast agent could be held in three directions with their cross‐sections in the coronal, sagittal, and axial planes, respectively, within the air cavities. MR images of the phantom were acquired using fast spin echo (FSE) and fast gradient echo (fGRE) sequences. The positions of the vials according to their centers of mass were measured from the MR images and registered to the corresponding computed tomography images for comparison. Results showed the fGRE sequence exhibited no errors >2.0 mm in the sagittal and coronal planes, whereas the FSE sequence produced images with errors between 2.0 and 4.0 mm along the phantom's perimeter in the axial plane. On the basis of these results, the fGRE sequence was considered to be clinically acceptable in acquiring images in all sagittal and coronal planes tested. However, the spatial accuracy in periphery of the axial FSE images exceeded the acceptable criteria for the acquisition parameters used in this study.

PACS number(s): 87.57.–s, 87.61.–c

## INTRODUCTION

Interest in the assessment of respiration‐induced lung tumor motion has increased with the advent of gated therapy.^1^ ^,^ ^2^ Information about the characteristics of tumor motion is important in the development and implementation of the gating techniques. Complementary to *x*‐ray computed tomography (CT), magnetic resonance (MR) imaging offers unique capabilities for tracking the lung tumor with flexible imaging planes, e.g., along sagittal or coronal planes with a large field‐of‐view (FOV) and with sub‐second temporal resolution.^3^ ^,^ ^4^ In this paper, we exploited some of the features of MR imaging using two pulse sequences: a cine imaging sequence designed for tracking lung tumors during respiration and a sequence designed for tumor identification and visualization.

Although the advantages of MR for visualizing soft‐tissue anatomy are well established, the use of MR imaging for radiation therapy treatment planning is frequently accompanied by concerns regarding the geometrical accuracy of the images. Spatial distortions of MR imaging may be either object‐induced or machine‐dependent. Some object‐induced distortions can originate from differences in the magnetic susceptibility of materials and from differences in the local electrochemical environment of the protons, e.g., chemical shift effects. Localized fluctuations in the magnetic field originating from large susceptibility differences can cause distortions near air‐tissue interfaces that can cause changes in the shape and location of the objects. Machine‐dependent errors are inherent and vary for each MR scanner. They are also systematic and appear in every image. Some examples of the causes of these errors include inhomogeneities in the main magnetic field, nonlinearities in the gradients, and eddy currents.

Because the thoracic region contains a large amount of air‐tissue interfaces and typically requires a large FOV, the magnitude of the MR image distortions may be particularly troublesome. Therefore, the aim of this study is to quantify the geometric distortion in the two image sequences mentioned above and to determine whether these images satisfy the spatial accuracy requirement for radiation therapy purposes (e.g., 2.0 mm). In the following sections, we will present the theoretical simulation of the magnetic susceptibility effect. This simulation is intended to predict the systematic shift in voxel position caused by susceptibility differences that cannot be measured with the image registration technique used here. Separate experiments were designed to measure the distortion of the thoracic MR images.

## METHODS

### A. Theoretical simulation

Differences in the magnetic susceptibility, $\Delta_{\chi }$, between two materials creates microscopic perturbations in the magnetic field at the interface of the materials. In the human body, interfaces of air (χ∼0.36 ppm) and tissue (χ∼11.0 to −7.0 ppm) give rise to the largest natural magnetic susceptibility field perturbations.^5^ The distortion is also dependent on the shape of the interface or object and the orientation of its axis with respect to the direction of the main magnetic field. For a cylinder with a circular cross‐section, the magnetic disturbances can distort and displace the cross‐section^5^ However, the distortion only occurs in the frequency‐encoding (FE) direction because the MR signal is sampled while the FE gradient is turned on. Therefore, no susceptibility distortions appear in the phase‐encoding (PE) direction.

**Figure 1:**
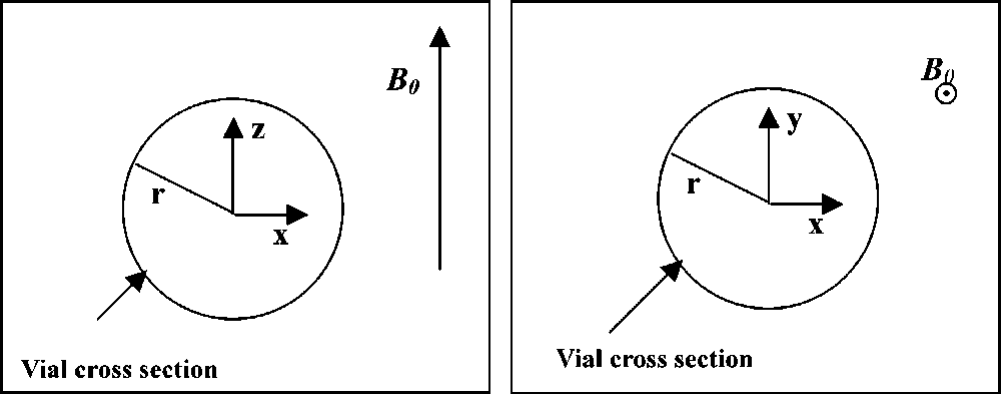
A schematic view of two situations simulated and measured. Situation 1 appears in the panel on the left and situation 2 in the panel on the right.

In this work, circular cross‐sections of vials with their axes oriented perpendicular and parallel with the direction of the main magnetic field were taken. The distortion experienced in the images of these cross‐sections can be predicted theoretically as detailed by Schenck.^5^ Figure 1 shows a schematic view of two situations: (i) a cylinder has its axis aligned with the *Y* direction, which is perpendicular to $B_{0}$, or the *z* direction, i.e., the cross‐section of the vial is in the *x‐z* plane; (ii) a cylinder with its axis parallel to the $B_{0}$, with the vial cross‐section in the *x‐y* plane.

For situation 1 (left), the change in the local magnetic field due to the susceptibility effect, $\Delta B_{z}$, just outside the vial, is described by Schenck,

(1) $$\Delta B_{z}(x,z)=\frac{\Delta_{\chi }B_{0}r^{2}}{2}\frac{z^{2}-x^{2}}{{\left(x^{2}+z^{2}\right)}^{2}},$$

where *r* is the radius of the vial, *z* is the distance from the vial center in the *z* direction, and *x* is the distance from the vial center in the *x* direction.^5^ $\Delta B_{z}$ inside the vial is given as

(2) $$\Delta B_{z}=\frac{\Delta \chi B_{0}}{2}.$$

For situation 2, Eqs. (1) and (2) reduce to $\Delta B_{z}=\Delta \chi \;B_{0}$ and $\Delta B_{z}=0$, respectively. The positional error created by $\Delta B_{z}$ is simply $\Delta B_{z}$ divided by the readout gradient magnitude $G_{r}$, which is

(3) $$G_{r}=\frac{BW}{\gamma FOV},$$

where BW is the bandwidth, *γ* is the gyromagnetic ratio, and FOV is the field‐of‐view. Thus, the position error can be calculated as,

(4) $$x\prime (x,z)=x+\frac{\Delta B_{z}(x,z)}{G_{r}}$$

For a known material imaged, such as water or tissue, this equation relates the possible shift, caused by differences in susceptibility, to the bandwidth and FOV used for the imaging. The theoretical prediction provides us with a guideline on the expected magnitude of the image distortion caused by the MR susceptibility effect alone. Based on these data, we simulated the distortion for whole deoxygenated blood (χ∼7.90 ppm) surrounded by air as is the situation in the lungs. The results will be presented in the Results section.

### B. Phantom experiment

**Figure 2:**
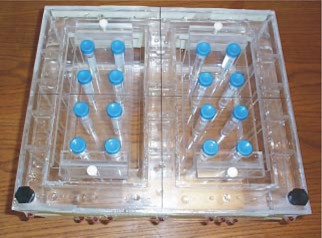
(Color) A view of the phantom with the inserts in place and vials loaded for coronal imaging.

A phantom was designed and built in‐house to approximate the geometry of the upper thorax, including two air cavities that served as simulated lungs (Fig. 2). The phantom size was 35×41×17 cm^3^, which was expected to enclose the thoracic region of most patients. The large size was intentional, because it required a large FOV, a condition under which image distortion from nonlinear gradients and magnetic field inhomogeneities was expected to be at its worst. Each air cavity of the phantom measured 28×15×15 cm^3^ and contained the inserts. The inserts were designed to hold the vials in place so vial cross‐sections could be imaged in the sagittal and coronal planes. Circular cross‐sections in the axial plane were obtained using the sagittal inserts and rotating the phantom 90° in the appropriate direction, which set the vial axes perpendicular to the axial plane. The inner diameter of the vials was about 1.5 cm. The size of the vial chosen was based on the size of a typical small lung nodule and the ability to image the vial with reasonable MR image resolution. The distance between two adjacent vials was approximately 3.8–5.6 cm depending on the arrangement. Filling the phantom and vials, except the air cavities, was 8.3 L of a solution consisting of deionized/degassed water, 2.4 g/L of NaCl, and 18 mL of a Gd‐DTPA doping agent (Magnevist™, Berlex Laboratories, Wayne, NJ). The solution had a measured T1/T2 of 204/112 ms.

MR images were acquired of the phantom on a 1.5‐T whole‐body Signa EchoSpeed MR scanner (v8.3, GE Medical Systems, Milwaukee, WI). An fGRE sequence was used to acquire images at selected slice locations. These images were obtained using the following parameters: $\text{BW}=83.3\;\text{kHz},\;\;\text{FOV}=44\;\text{cm},\;\;\text{TE}/\text{TR}=2/4\;\text{ms},\;\;\text{flip}\;\text{angle}=60^{\circ },\;\;\text{NEX}=3.0,\;\;\text{matrix}=512\times 512$, and slice thickness of 1.0 cm. These sequence parameters were chosen previously to image the lung and to track the tumor motion with optimized tumor contrast, except here the image matrix was increased from 256×256 for the patient scan to 512×512 for the phantom scan in order to increase the spatial resolution.

**Figure 3:**
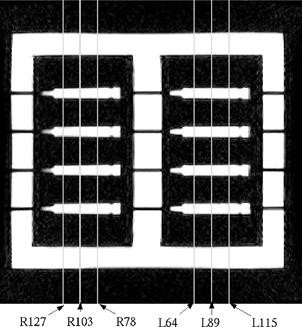
Labeled sagittal plane locations taken of the phantom with sagittal inserts in place. The sagittal scan planes, in combination with these inserts, produced circular cross‐sections of the vials seen in the subsequent figures.

For the sagittal fGRE sequence, the vials were oriented left and right. Six sagittal slice locations were prescribed, three per air cavity. The center of the phantom in the right‐to‐left (RL) and superior‐to‐inferior (SI) directions was landmarked with the sagittal and axial alignment lights, referred to as R0 and S0 in this study, respectively. For example, R127 corresponds to a slice in the sagittal plane centered 12.7 cm right of the phantom's center. All six sagittal locations are shown in Fig. 3. The anterior‐to‐posterior (AP) direction of the phantom was designated as the PE direction, and the SI direction of the phantom was along the FE direction.

Two coronal plane images, located at A33 and A13 where A0 corresponds to the center of the magnet along the AP direction, were acquired using the same fGRE sequence. For these scans, vials were inserted as shown in Fig. 2. The PE and FE directions were in the SI and RL directions, respectively.

**Figure 4:**
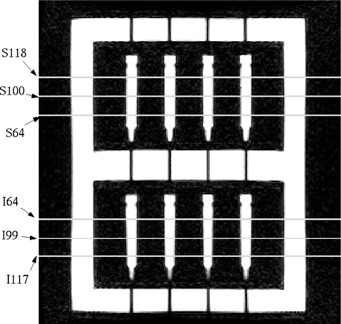
A coronal view of the phantom when it was rotated for taking axial images with inserts in place. The plane locations where the axial images intersected the volume are labeled.

The second sequence tested was a T1‐weighted fast spin echo (FSE) sequence used to acquire the axial images of the lung and identify the lung tumor. The acquisition parameters were: BW=62.5 kHz,  TE/TR=9.4/600 ms,  echo  train  length=3,  FOV=44 cm,  NEX=3.0,  and  image  matrix=512×512. Five axial images were taken at each of six locations (S118, S100, S64, I64, I99, and I117 mm) shown in Fig. 4. The labeling is similar to that of the sagittal images in Fig. 3 but involves the superior (S) and inferior (I) directions. To test the image accuracy at extended distances from the magnet's center, all the scan planes were acquired in the same series. Thus, each slice was not centered at the isocenter of the magnet during image acquisition. This setup was different from acquiring the sagittal and coronal images, for which each slice was located at the center the main magnet along the SI direction.

To allow for measurement of the spatial accuracy of the MR images of the phantom, x‐ray CT images were acquired on a multislice helical CT scanner (LightSpeed QX/^*i*^ General Electric Medical Systems) and used as the standard for comparison. Because CT only allows for axial image acquisition, the phantom was rotated appropriately during the scan to match with those imaging planes (sagittal and coronal planes) used for the MR imaging of the phantom. A spirit level was always used to level the phantom and avoid oblique slices. Then, every MR image acquired was paired with its corresponding CT image. Each pairing of CT and MR images constituted an image set to allow for image registration to be described next.

First, the MR and CT images were visually inspected and compared to determine whether significant image distortion to the geometrical shape and dimensions of all the phantom compartments and the vials appeared in the MR images. To quantitatively measure the MR image distortion within the air cavities, the positions of the vials in the images were used as spatial landmarks in comparing the MR versus CT images. In this process, the coordinates of the centroid of every vial cross‐section in the image sets were calculated. This calculation required a defined region of interest (ROI) surrounding each vial, within which the maximum pixel value was determined. The outer boundary of the vial cross‐section was determined by setting to zero pixel values that were less than or equal to the half‐maximum value in the ROI surrounding the vial. Then, the center‐of‐mass (COM) coordinates $\left(x_{com},y_{com}\right)$ of each vial in the image was calculated using the remaining nonzero pixels and definitions,

(5) $$\begin{array}{l} x_{com}=\frac{\Sigma x_{i}}{N},\\ y_{com}=\frac{\Sigma y_{i}}{N}, \end{array}$$

where $x_{i}$ and $y_{i}$ are the coordinates of the individual pixels within the vial boundary and *N* is the total number of pixels within the vial boundary.

To register an MR image with its corresponding CT image, a new coordinate system common to both the images was defined. This was accomplished by first choosing a registration point from the COM of one of the vials as centrally located in the image as possible. This registration point subsequently became the origin of the common coordinate frame. (See Fig. 7) Next, using the least squares method, a common *x* axis was fit through a row of vial centroids including the registration point. The *Y* axis was set perpendicular to the *x* axis and passed through the registration point. The rotation angle of the object in the image was determined from the slope of the common *x* axis using, i.e., $\theta =\arctan(m_{0})$, where $m_{0}$ is the slope of the fit *x* axis to the object. Then, a matrix transformation registered the COM coordinates in their original Cartesian image frame to the common coordinate frame,

(6) $$[x\prime \;y\prime \;1]=[x\;y\;1]*\left[\begin{array}{ccc} \cos(\theta ) & \sin(\theta ) & 0\\ -\sin(\theta ) & \cos(\theta ) & 0\\ 0 & 0 & 1 \end{array}\right]\frac{FOV}{MATRIX},$$

where (FOV/MATRIX) scales the object coordinates to centimeters.

With the COM coordinates computed in their new registered frame, each vial centroid position in the MR and CT images was compared. The differences in the coordinates along the PE and FE directions were compiled separately for each image set. However, the displacement in the FE direction caused by susceptibility differences cancels out when the vials in the respective images are used as the bases for registration. Therefore, the theoretical simulation supplements the phantom measurements. The relevancy of this displacement to the forecasted uses of the MR sequences will be discussed further in the Discussion section of this paper.

Fig. 6. The displacement due to magnetic susceptibility differences between whole blood and air. This illustrates the case for circular cross‐sections of vessels in sagittal or coronal images.

## RESULTS

### A. Theoretical simulation

**Figure 5:**
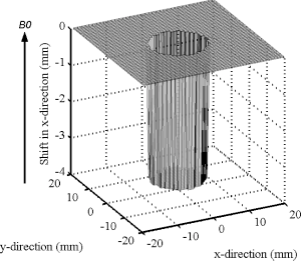
The displacement due to magnetic susceptibility differences between water and air. The case when the circular cylindrical axes were parallel to the main field.

The positional shift in the FE direction created by susceptibility differences between the whole blood and air is illustrated in Fig. 5. This figure illustrates the case where the blood vessels are parallel to the magnetic field, as the circular cross‐sections of vessels would be during axial image acquisition. Here, $\Delta B_{z}=\Delta \chi \;B_{0}\;\text{and}\;\Delta B_{z}=0$, are used in Eqs. (3) and (4) with the parameters $B_{0}=1.5\;T,\;\;\Delta \chi =-7.90,\;\;\text{BW}=62.5\;\text{kHz},\;\;\text{FOV}=44\;\text{cm}$, and γ=42.58 MHz/T. The theoretical maximum displacement due to susceptibility differences alone is −3.7 mm.

**Figure 6:**
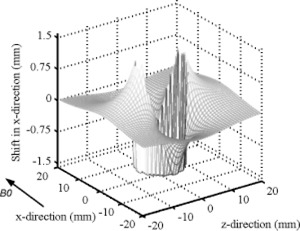
A CT image (at left) and a sagittal MR image (at right) of the phantom. The directions of the PE and FE, the index for the columns, and rows of the vials are shown.

Figure 6 shows the shift when the vial axes are perpendicular to the magnetic field, as they were during coronal and sagittal image acquisition. Here, the results of Eqs. (1)–(4) are combined with the parameters $B_{0}=1.5\;T,\;\;\Delta \chi =-7.90,\;\;r=1.5\;\text{cm},\;\;\text{BW}=83.3\;\text{kHz},\;\;\text{FOV}=44\;\text{cm}$, and γ=42.58 MHz/T to generate this figure. The theoretically largest displacement is 1.4 mm.

It should be noted that it is the vials that are full of doped water, surrounded by air. Therefore, distortion of the cross‐sections does not manifest itself as an arrowhead shape. This is due entirely to the fact that the surrounding air gives no signal with which the nonuniform susceptibility perturbations that exist exterior of the interface can change. The result is a uniform shift of the signal existing interior to the material interface, i.e., a shift in the location of the cross‐section.

### B. Phantom experiment

**Figure 7:**
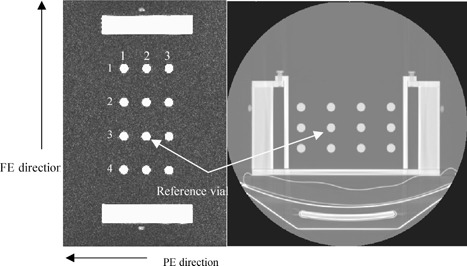
A CT image (at left) and a coronal MR image (at right) of the phantom. The results in Table III are arranged to correspond with the row and column indices shown in the MR image.

**Figure 8:**
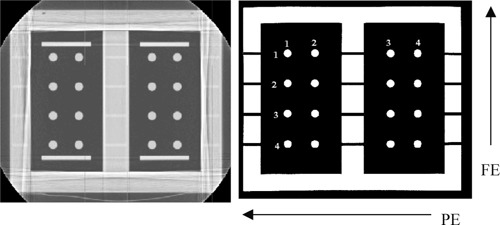
An axial MR image of the phantom. The corresponding CT image is similar to that shown in The reference vial, PE and FE directions, and column and row indices are labeled.

**Figure 9:**
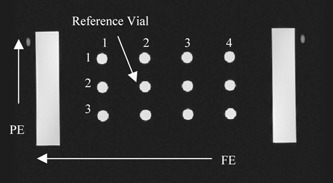
The differences in individual vial positions along the PE and FE directions in the sagittal fGRE images.

Examples of sagittal and coronal MR images acquired using the fGRE sequence and the axial MR images acquired using the FSE sequence, are shown in Figs. 7, 8, and 9, respectively. The CT images are also presented as a comparison, except for the axial MR series, which was able to use the same CT images as the sagittal series. The images demonstrated that the degree of distortion was not apparent in the fGRE images from the geometry and shape of the phantom compartment. In particular, the air‐tissue magnetic susceptibility effect did not cause a readily observable distortion on the vial cross‐sections. However, warping of the phantom edges did exist near the extreme edge of the phantom in the FSE sequence. The quantitative results are presented in the text and tables that follow.

**Table I:** The average absolute differences in vial positions from the sagittal fGRE MR images in the two most lateral slices (R127 and L115, see Fig. 3).

	Direction	Column 1 avg (mm)	Column 2 avg (mm)	Column 3 avg (mm)
Row 1	FE	0.6	0.4	0.2
	PE	0.6	0.7	0.5
Row 2	FE	0.4	0.3	0.4
	PE	0.3	0.3	0.6
Row 3	FE	0.1	0.0	0.1
	PE	0.1	0.0	0.4
Row 4	FE	0.8	0.3	0.3
	PE	0.5	0.1	0.3

The average differences in the vial positions along the PE and FE directions are shown in Tables I and II for the medial and lateral sagittal fGRE images. The results from two corresponding imaging planes in the two air cavities were averaged, i.e., the most lateral position is from the two imaging planes R127 and L115 and the most medial position is from the two imaging planes R78 and L64, (see Fig. 3). These two tables are arranged similar to the arrangement of the vials that appear in Fig. 7 to show the relative AP and SI location of the vials in the phantom. That is, the outer rows and columns contain the vials along the phantom's perimeter. Additional results for the mid‐cavity slices (R103 and L89, see Fig. 3) are not listed in a separate table here because they are similar to those shown in Table II. Our results indicated that the averaged absolute difference over all the vials was 0.4±0.3 mm for both the PE and FE directions for the outermost and mid‐cavity slices. Similarly, the averaged difference was 0.3±0.2 mm for the medial slices. The maximum absolute differences in the PE direction was 1.1 mm along the PE directions, located at row 1 column 3 (vial (1,3) near a corner) in a mid‐cavity slice. For the FE direction, the maximum absolute difference was 1.3 mm at vial (4,1), also near a corner, in a lateral slice. The positional difference for the reference vial was not included, because by default it was located at the origin of the common registration coordinate frame of the CT and MR images.

**Figure 10:**
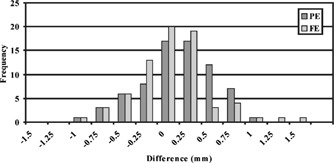
The differences in individual vial positions along the PE and FE directions in the coronal fGRE images.

Figure 10 shows a histogram of the vial displacements in all sagittal images. Notice the majority of displacements were within 1.0 mm, and their distribution exhibited a Gaussian behavior. The displacements are shown with their +/− signs, where (+) signs in the PE and FE directions indicated the vials appeared to the left or below of their corresponding vials in the CT images, respectively. Similarly, (–) signs in the PE and FE directions indicated the vials appeared to the right or above of their corresponding vials in the CT images, respectively. The larger differences tended to exist in the outer region, or lateral, slice locations.

**Table II:** The average absolute differences in vial positions from the sagittal fGRE MR images in the two most medial slices (R78 and L64, see Fig. 3).

Direction	Column 1 avg (mm)	Column 2 avg (mm)	Column 3 avg (mm)
Row 1	FE	0.4	0.3	0.2
	PE	0.3	0.4	0.4
Row 2	FE	0.2	0.2	0.3
	PE	0.3	0.2	0.4
Row 3	FE	0.2	0.0	0.1
	PE	0.3	0.0	0.2
Row 4	FE	0.7	0.4	0.2
	PE	0.5	0.1	0.1

**Figure 11:**
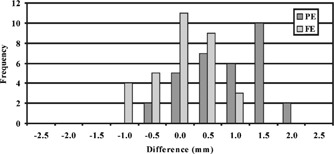
A histogram showing the frequency of differences that occurred between the positions of vials in axial FSE MR and CT images.

The results of the coronal fGRE images are shown in Table III. The absolute differences for each vial position averaged over the two coronal planes is shown in this table. The position difference from all the vial locations equaled 0.9±0.5 mm and 0.8±0.5 mm with the maximum of 1.7 and 1.2 mm along the PE and FE directions, respectively. Both of these maximum errors occurred on row 1 of the phantom at column 1 and 4 as pictured in Fig. 8, respectively. Figure 11 shows the histogram of the vial position differences. Again, the majority of the errors are within 1.0 mm with none exceeding 2.0 mm, and the larger differences occurred to the vials at the outer boundary.

For the axial FSE images, Table IV shows the differences in the vial positions averaged for the two medial axial slices. Notice that all of the vials except those in column 4 of Table IV, showed average absolute differences within 2.0 mm in the PE and FE directions (1.7±1.0 mm and 0.9±0.6 mm, respectively). Results of the two other pairs of mid‐cavity and outermost slices showed increased differences. Table V shows the outermost slices. Large differences are seen at the corner vial locations. The averaged absolute differences for all locations in the PE and FE directions were 1.7±1.0 mm and 1.2±0.7 mm, respectively. The maximum difference was 3.6 mm in the PE direction for a vial at row 1 and column 1 situated at the corner in an innermost slice. The results indicated that the differences along the PE direction showed no apparent dependency on imaging plane or vial location. However, the differences in the FE direction decreased for the vials closer to the magnet's center.

Figure 12 shows a cumulative histogram of the differences in all the vial positions (from all six imaging slices) in the MR axial FSE images. This histogram and our data analysis show that the position differences exceed 2.0 mm along the PE direction for 22 out of 66 vial cross‐sections or 33% of the vials excluding the reference vials. Seventeen of the 22 larger differences occurred on the left perimeter of the phantom in column 4. The rest occurred at vial (3,3) and vial (1,1) if looking at the matrix arrangement shown in Fig. 9. Along the FE direction, less than 5% of the vial locations showed differences larger than 2.0 mm, and the maximum position difference all occurred at the top left vial in column 4. There were two other vials at (2,4) and (3,1) that showed differences right at 2.0 mm. All vial displacements that equaled or exceeded 2.0 mm occurred along the periphery of the vial arrangement.

**Table III:** The average absolute differences in the vial positions from the two coronal slices (see Fig. 7).

	Direction	Column 1 avg (mm)	Column 2 avg (mm)	Column 3 avg (mm)	Column 3 avg (mm)
Row 1	FE	1.4	1.3	0.6	0.3
	PE	0.6	0.4	1.0	1.2
Row 2	FE	1.4	1.2	0.2	0.1
	PE	0.4	0.3	0.4	0.6
Row 3	FE	0.9	1.3	0.0	0.0
	PE	0.4	0.3	0.0	0.1
Row 4	FE	1.3	1.0	0.2	0.7
	PE	0.5	0.5	0.4	0.3

## DISCUSSION

Geometrical accuracy of MR images has long been an issue of concern, particularly for radiation therapy applications.^6^ ^,^ ^7^ In this work, the spatial accuracy of the MR images was determined for the specific image sequences to be used to assess lung tumor and its motion. The fGRE images of the thoracic phantom demonstrated an acceptable level of spatial accuracy, because the displacements measured did not exceed 2.0 mm. The fidelity of these fGRE images could be mainly contributed by the fact that the systematic distortion was minimum near the center of the magnetic field of this particular scanner, even with a large FOV of 44×44 cm^2^. In addition, because of the high bandwidth used for the imaging (83.33 kHz), the susceptibility effect was estimated to be about 1.5 mm within the vials, which did not cause significant artifact for the vial image apparently. This measurement is consistent with previous studies reporting on the use of MR and CT image registration for radiation therapy treatment planning. Turkington *et al*.^8^ reported translational errors less than 2 mm in the registration of head‐phantom images. Other reports are in agreement with these findings, reporting errors between 0.3 and 2.2 mm.^9^ ^,^ ^10^

The results from the FSE axial images showed a larger degree of discrepancy exceeding 2.0 mm. These larger differences occurred in the PE direction for 33% of the vial locations tested, most of which occurred in column 4 as depicted in Fig. 9. The cause of the greater difference in the FSE axial images may be attributed by the fact that these axial FSE images were acquired with the imaging planes located away from the magnet center. Thus, the systematic errors from inhomogeneity of the main magnetic field and gradient nonlinearity may be more pronounced at these off‐center locations.

**Table IV:** The average absolute differences in the vial positions in the most medial axial slices (see Fig. 8).

	Direction	Column 1 avg (mm)	Column 2 avg (mm)	Column 3 avg (mm)	Column 4 avg (mm)
Row 1	FE	0.6	1.0	1.5	2.1
	PE	1.9	0.3	1.1	2.6
Row 2	FE	0.3	0.0	0.5	1.0
	PE	1.5	0.0	1.5	3.0
Row 3	FE	1.3	1.0	0.3	0.3
	PE	1.2	0.3	1.9	3.5

**Table V:** The average absolute differences in the vial positions in the most distal and proximal axial slices (see Fig. 8).

	Direction	Column 1 avg (mm)	Column 2 avg (mm)	Column 3 avg (mm)	Column 4 avg (mm)
Row 1	FE	1.0	1.1	1.6	2.5
	PE	1.9	0.4	0.9	2.3
Row 2	FE	0.9	0.0	0.6	1.4
	PE	1.4	0.0	1.4	2.9
Row 3	FE	1.3	1.0	0.6	0.8
	PE	1.2	0.5	2.1	3.4

In registering the MR and CT images, a common coordinate frame was established with an origin selected near the center of the image. The rationale of such an approach is that the image distortion is minimum near the center of the magnetic field. Though the differences in the vial positions between the MR and CT images could be subject to the specific location of the reference point, we expect that the effect of choosing the reference point would be negligible as long as it is near the center of the magnet and FOV. In addition, the position differences measured from the fGRE images were all significantly less than 2 mm. Thus, selecting a more central reference point would not invalidate our results.

Registering the images in this way eliminates systematic shifts in the MR images. Such shifts take place due to differences in susceptibility as described in detail above. Because the shift is systematic, it is sufficient to ignore them for our usage of these images in radiation therapy applications. Our use of the images will include only relative measurements of the tumor or lung position change to be compared with the relative change in position of the skin surface or diaphragm.

The results presented above are specific to the MR sequences and the scanner used in this work. However, the cine fGRE sequence used for measuring patient tumor and lung motion required a 256×256 matrix size and three‐quarter echo acquisition (NEX=0.75) to improve the temporal resolution to less than 0.5 s/image. The increase of the voxel size will help to reduce the shift in the number of voxels caused by the image distortion, the basic principle of which has been shown by Eqs. (3) and (4). Thus, we do not expect that the magnitude of the image distortion will exceed those measured from the phantom experiments. For other radiation therapy imaging needs, the spatial accuracy of the MR images can be established in a fashion similar to what has been presented here, although the results should be expected to be unique to individual applications and MR scanners.

## CONCLUSIONS

In this work, the geometrical accuracy of the MR images was evaluated for the MR sequences used to assess the lung tumor motion. A thoracic phantom was built with two lung cavities, each filled with a grid of vials that were located in different regions of the cavities. Sagittal and coronal MR images were acquired using a fGRE sequence, whereas axial MR images were acquired using an FSE sequence. MR and CT images were registered with respect to a common reference point in each image. Following this, the positional differences of the vials in the MR and CT images were calculated. These results showed that the vial positions in the sagittal and coronal fGRE images had displacements no greater than 2.0 mm in either the FE or PE direction compared with those from the CT images. However, discrepancies exceeding the acceptable limit of 2.0 mm were found along the periphery in the axial images using the FSE sequences. The vast majority of these larger errors occurred in the PE direction, possibly due to the systematic distortion that was greater at the imaging planes away from the magnet center. The spatial accuracy for the sagittal and coronal images were found to be acceptable for subsequent patient imaging, whereas additional assessment for the axial image has to be made to improve their spatial accuracy for radiation therapy applications.

Images used in this study were acquired on an older model scanner with a long bore magnet. However, the recent trend in clinical images has been towards shorter bore magnets, which affect the length of the gradient coils. In turn, the manufacturer must contend with an increase in the magnitude of uncorrected errors near the field edge due to gradient nonlinearity. These errors, as are the errors in the older and longer bore magnets, are corrected for by unwarping during image reconstruction. Therefore, the end‐user of the short bore MR imaging units needs to be aware of the increased likelihood of seeing larger distortions near the field edge, if inadequately corrected.
